# Effect of single session receptive music therapy on anxiety and vital parameters in hospitalized Covid-19 patients: a randomized controlled trial

**DOI:** 10.1038/s41598-022-07085-8

**Published:** 2022-02-24

**Authors:** Filippo Giordano, Antonia Losurdo, Vitaliano Nicola Quaranta, Nicla Campobasso, Antonio Daleno, Elisiana Carpagnano, Loreto Gesualdo, Antonio Moschetta, Nicola Brienza

**Affiliations:** 1 Department of Emergency and Organ Transplants, University of Bari Aldo Moro, Piazza G. Cesare 11, 70124 Bari, Italy; 2 University of Bari Aldo Moro, Bari, Italy; 3 Pneumology Department, “Di Venere” Hospital Bari, Bari, Italy; 4 Azienda Universitaria Ospedaliera Consorziale-Policlinico Bari, Bari, Italy; 5 Department of Basic Medical Science, Neuroscience, and Sense Organs, University of Bari Aldo Moro, Bari, Italy; 6 Department of Interdisciplinary Medicine, University of Bari Aldo Moro, Piazza G. Cesare 11, 70124 Bari, Italy

**Keywords:** Psychology, Health care, Medical research

## Abstract

Hospitalized COVID-19 patients are vulnerable to different degrees of stress disorders as well as depression, anxiety and fear. The aim of this study was to evaluate the feasibility of introducing Music therapy on site with Covid-19 patients and investigating the immediate effects a single session has on anxiety, heart rate (HR), oxygen saturation (O2Sat) and satisfaction compared to standard care. A randomized controlled trial of 40 patients was conducted. Participants were assigned to control group (CG) or the treatment group (MG). MG received an individual single session of music therapy in presence. CG received standard care. MG and CG were subjected to identical measurements (pre-during-post) of the parameters STAI-Y, HR and O2Sat. Participants in MG were asked to fill in an optional open-ended question concerning their experience with music therapy. Significant difference in anxiety levels between scores in MG and CG (34.50 (23.25–40.00) vs 45.00(38, 25–54.00); p = 0.000) was observed. MG compared to CG had statistically significantly higher values of O2Sat (97.50 (96.25–99.00) versus 96.00 (96.00–98.00); p = 0.026). Results show the feasibility of introducing music therapy as a supporting complementary/non-pharmacological intervention on site in Covid-19 patients. A single session of music therapy improves O2Sat and can significantly reduce anxiety.

Trial registration: 14/10/2021 No. NCT05077306. https://www.clinicaltrials.cov.

## Introduction

The outbreak of the coronavirus disease (COVID-19) has caused great public concern^1^, unprecedented challenges to health care systems and huge psychological distress^2^.

Hospitalized patients are extremely isolated from their families for a long and uncertain period of time^3^. They remain in an undefined mental space left to wonder if this is a temporary separation or a step towards death^4^ which could take place without family or loved ones by their side^5^. This traumatic separation makes patients vulnerable to different degrees of stress disorders as well as depression and anxiety^6^, fear of the unknown and dying, sleeplessness, agitation, discomfort, pain, immobility, frustration and inability to relax^7^.

Music can be used as complementary/non-pharmacological intervention to reduce anxiety and stress during hospitalization in different ways.

Music Therapy is defined as the systematic use of musical experiences aimed at achieving therapeutic goals by a trained music therapist and implies the establishment of a relationship between patient, music and Music therapist, while Music Medicine (MM) is considered as passive listening to pre-recorded music provided to the patient by a nurse or other medical staff^8^.

Music therapy has been shown to play a valuable role in the care of patients with serious illness, helping to address physical symptoms and psychological distress^9^. Music therapy reduces pain, improves sleep quality, decreases anxiety during mechanical ventilation, induces a relaxation response without the use of medication (Mofredj et al. 2016), lowers the respiratory rate and blood pressure, and has a possible positive impact on the use of sedatives and analgesics among mechanically ventilated patients^10^. To date, however, there are no studies of music therapy application on-site with Covid 19 patients.

The primary aim of this study was to evaluate the feasibility of introducing music therapy on site with Covid 19 patients as a supporting complementary/non-pharmacological intervention.

The secondary aim was to investigate the immediate effects a single music therapy session has on anxiety, heart rate (HR), oxygen saturation (O2Sat) and satisfaction compared to standard care.

## Methods

### Participants

Over a 4-week period (from 15th April to 15th May 2021), all patients with SARS-COV2, hospitalized at Covid Hospital Bari, Italy were screened in the study.

Exclusion criteria were (a) age ≤ 18 years, (b) severe neurological or psychiatric conditions, (c) hearing impairment, (d) intubation.

Study protocol was approved by the Hospital Ethics Committee of Bari, (no. 6841-09/04/2021) and patients signed an informed consent form.

All methods were performed in accordance with the relevant guidelines and regulations.

## Design and procedure

A mixed-methods approach pre -post design was used to obtain and evaluate data in 2 areas: (1) the feasibility of delivering music therapy on site in the Codiv19 hospital and (2) the immediate effects of the intervention on patients’ HR, O2Sat, anxiety and satisfaction.

A sample size of 20 per group (N = 40) was calculated to achieve 80% power to detect a difference of 11.0 with a SD (standard deviation) of 12.5 in anxiety score. The sample size was calculated based on the results of a previously published trial^11^.

This study employed a patient-centered approach in which the music therapist tailored interventions to patients’ individual needs in that moment (Bradt et al. 2016). An interactive relational approach of receptive music therapy (Bruscia 1998a; Grocke and Wigram 2007), supplemented by adaptation of the Bonny Method Guided Imagery and Music in the medical setting (MED-GIM)^12^ was used (Bruscia and Grocke 2002)^13^.

Participants were assigned to control group (CG) or treatment group (MG) by computer simple randomization. Participants in MG received an individual bedside single session of receptive music therapy by a certified music therapist-GIM fellow in presence. Each session consisted of five parts: (1) patient assessment and verbal interaction to help the patient to disclose a concern, facilitating or encouraging a state of mind of wellbeing or enjoyment that is known to the patient^13^; (2) creation of customized play list by music therapist following both patient's assessment and the specific music elements as pulse, mood, melodic line, dynamic change, bass line, volume, timbre, rhythm, form; (3) brief relaxation exercise to help patients find an image as the focus with a positive outcome; (4) music listening, with dialogue between patient and music therapist; (5) conclusion to validate feelings and reinforce a positive experience^14^.

Participants listened to the playlist with bone conduction headphones from Ipod® and volume was controlled by the music therapist. The music therapist tailored music for each participant was based on the results of patient assessment (Robb, Carpenter, & Burns, 2011). The music therapist used music selected from classical music of the Western tradition, pop, rock, new age, soundtrack, light jazz CG received standard care.

This study was designed in accordance with the Consolidated Standards of Reporting Trials (CONSORT) recommendation for RCTs.

### Measure

State Trait Anxiety Inventory Y-1(STAI-Y1) was used to measure how the subject felt in that moment. Subjects were asked to rate the intensity of their anxious feelings on 20 items on a four point scale: not at all, somewhat, moderately so, or very much so.

MG and CG were subjected to identical measurements of the parameters STAI-Y, HR and O2Sat.

STAI-Y values < 40 defined absence of anxiety, between 40 and 50 mild anxiety, 51–60 moderate anxiety, and > 59 severe anxiety.

From the variables under study the parameter ΔSTAI-Y was derived, defined as the difference between the value of STAI-Y calculated at time T2 minus the value of STAI-Y calculated at time T0. Similarly, the parameters ΔO2Sat% and Δ H.R. were calculated. ΔO2Sat% was defined as the difference between the value of O2 Sat calculated at time T2 minus the value of O2Sat at time T0. ΔHR was defined as the difference between the value of O2Sat calculated at time T2 minus the value of O2Sat at time T0. STAI Y-1 was administered 5 min before session (STAI Y-1 PRE) and 15 min after session (STAI Y-1 POST) in paper form in MG and CG.

HR and O2Sat were recorded from the bedside monitor three times: start session (T0), 10 min. (T1), end session (T2). At the same time, STAI Y-1, O2Sat and HR were recorded in CG. Participants in MG were asked to fill in an optional open-ended question concerning their experience with RMUSIC THERAPY. HR and O2SAT were collected by music therapist, STAI Y-1 and optional open-ended questions were collected by a psychologist researcher.

### Statistical analysis

Continuous variables were expressed as Mean ± SD and median (IQ25, IQ 75) depending on whether they were with normal or non-normal distribution. The dichotomous or non-continuous variables were expressed as%. We verified the non-normal distribution of the continuous variables under study using the One Sample Kolmogorov–Smirnov test.

The dichotomous variables were compared with the Chi Square test.

Variables with normal distribution were compared with the Student's T-test for independent samples and variables with non-parametric distribution were compared with the Mann–Whitney U test. Non-parametric analysis was carried out by means of the Friedman test, and subsequently, if a significance emerged, the two-by-two comparison between the times was carried out with the Wilcoxon test.

To correctly classify the MG with the control group, linear canonical discriminant analysis was used to create a model that optimizes the between sample classes and within-sample class distances. The cross validated accuracy percentage (CVA, %) was calculated.

All analyzes were conducted with the SPSS 23 software. Statistical significance was assumed for p value < 0.050.

## Results

Forty subjects were randomized into two homogeneous subgroups of 20 patients each. Patients were divided into the 2 groups in order to have statistically similar values of age, sex and clinical parameters P/F ratio and use of NIV/CPAP.

Patients in MG compared to CG had comparable values of age, sex, P/F ratio (300.56 ± 101.89 vs 267.40 ± 94.65; p = 0.293), use of CPAP or NIV (15% vs 20%; p = 0.500) (Table1).

**Table 1:** Comparison of music therapy group (MG) versus control group (CG).

	MG n = 20	CG n = 20	P value
Age, M ± DS	57.63 ± 9.00	61.35 ± 9.41	0.215
Sex F %	55.00	25.00	0.100
P/F ratio, M ± DS	300.56 ± 101.89	267.40 ± 94.65	0.293
NIV/CPAP yes %	15.00	20.00	0.500
STAI-y value pre, M ± DS Median (IQ 25, IQ 75)	45.95 ± 14.52 42.00 (38.25–60.50)	44.80 ± 10.42 45.00 (36.75–54.00)	0.775
SATI-Y < 40 pre %	35.00	35.00	0.629
Low SATI-Y pre %	30.00	20.00	0.358
Intermediate STAI-Y pre %	10.00	45.00	**0.015**
high STAY_Y pre %	25.00	0.00	**0.024**
STAI value post, M ± DS Median (IQ 25, IQ 75)	33.60 ± 9.54 34.50 (23.25–40.00)	46.15 ± 9.16 45.00 (38.25–54.00)	**0.000**
STAY < 40 post %	70.00	35.00	**0.028**
LOW STAI-Y post %	30.00	20.00	0.358
Intermediate STAI-Y post %	0.00	40.00	**0.002**
high STAY_Y post %	0.00	5.00	0.500
FC pre M ± DS Median (IQ 25 -IQ 75)	81.05 ± 12.38 78.00 (72.25–88.75)	80.50 ± 14.28 78.50 (73.50–82.75)	0.897
FC during, M ± DS Median (IQ 25–IQ 75)	75.25 ± 11.05 73.00 (68.50.00–84.50)	81.05 ± 14.14 79.50 (74.25–82.75)	0,157
FC post, M ± DS Median (IQ 25–IQ 75)	75.40 ± 10.83 73.50 (67.50–85.00)	80.95 ± 13.83 78.50 (73.75–83.00)	0,166
O2 Sat pre, M ± DS Median (IQ 25–IQ 75)	96.15 ± 1.78 96.50 (95.00–97.75)	96.70 ± 1.38 97.00 (96.00–98.00)	0.283
O2Sat during session, M ± DS Median (IQ 25–IQ 75)	97.95 ± 1.35 98.00 (97.00–99.00)	96.60 ± 1.23 96.00 (96.00- 98.00)	**0.002**
O2Sat post, M ± DS Median (IQ 25–IQ 75)	97.65 ± 1.30 97.50 (96.25–99.00)	96.65 ± 1.42 96.00 (96.00- 98.00)	**0.026**
ΔO2 Sat%, M ± DS Median (IQ 25, IQ 75)	1.50 ± 1.00 1.50 (1.00–2.00)	−0.050 ± 0.60 0.00 (0.00–0.00)	**0.000**
**Δ**STAI-Y, M ± DS Median (IQ 25, IQ 75)	−12.35 ± 8.61 −13.00 (−17.00 to −3.25)	1.35 ± 2.90 0.00 (0.00–0.00)	**0.000**
**Δ**H.R, M ± DS Median (IQ 25, IQ 75)	−5.65 ± 1.19 −4.50 (−8.00 to −2.00)	0.45 ± 1.27 0.00 (0.00–1.00)	**0.000**

*P/F* ratio PaO2/FiO2, *STAI-y* STAI-y: State-Trait Anxiety Inventory—y form, *H.R*. heart rate, *O2Sat* oxyhemoglobin saturation, ***Δ****O2 Sat%* difference between the value of O2 Sat calculated at time T2 minus the value of O2 Sat at time T0, *TO* time at the start of the session, *T1* time during the session, *T2* time at the end of the session, *ΔH.R*. difference between the value of O2 Sat calculated at time T2 minus the value of O2 Sat at time T0, ***Δ****STAI-Y* difference between the value of STAI-Y calculated at time T2 minus the value of STAI-Y calculated at time T0.

Significance was assumed for p values < 0.050.

### Comparison between group

#### TO

From the comparison analysis between groups it was verified that the subjects in MG compared to the CG immediately before the start of the session had similar medians of STAI-Y values with similar percentages of absence of anxiety and mild anxiety. They had higher rates of severe anxiety values (25% vs 0%; p = 0.024) and lower rates of intermediate anxiety values (10% vs 45%; p = 0.015). MG compared to CG also had similar median values of O2Sat and HR.

#### T1

After 10 min from the beginning of the session, the patients in MG compared to those of CG had similar values of HR. It also emerged, however, that in MG the O2Sat was slightly, yet significantly, higher (98.00 (97.00–99.00) versus 96.00 (96.00–98.00); p = 0.026).

#### T2

At the end of the session, it emerged that the medians of STAI-Y of the subjects who carried out a music listening session compared to the others showed significantly lower values of anxiety (34.50 (23.25–40.00) vs 45.00 (38, 25–54.00); p = 0.000) and at this time 70% had no anxiety, 30% had low anxiety values and none had moderate or severe anxiety values. In subjects of CG, on the other hand, 35% did not show anxiety, 20% showed low values, 40% moderate values, and 5% severe values. Similarly, MG compared to CG had statistically significantly higher values of O2Sat (97.50 (96.25–99.00) versus 96.00 (96.00–98.00); p = 0.026).

Finally, the patients undergoing receptive music therapy presented similar values of HR.

### Intra-group comparison between the times T0, T1 and T2

**Figure 1:**
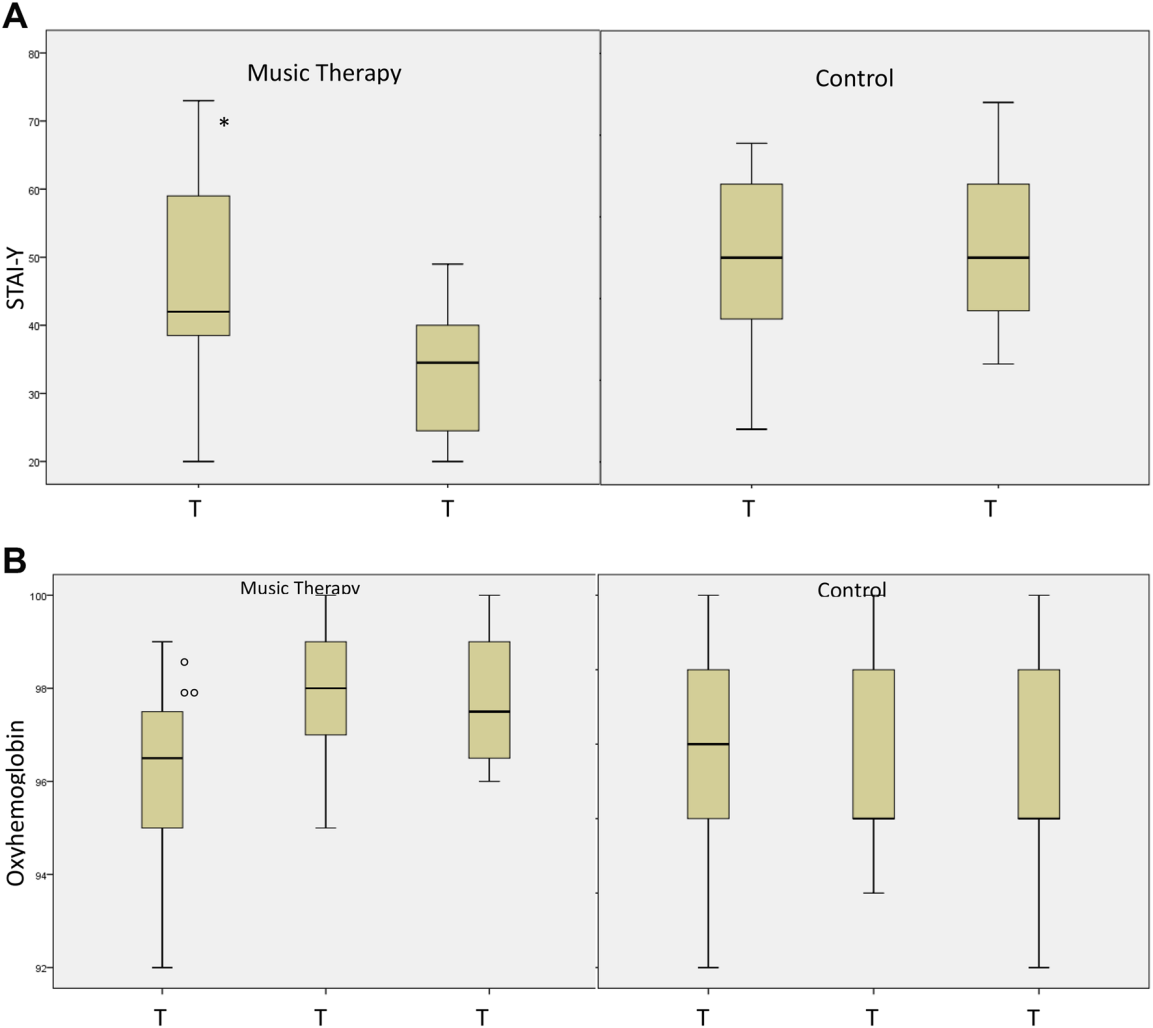
Comparison box plot at different times of the MG and of the CG for the STAI-Y parameters and percentage of Oxyhemoglobin Saturation. *STAI-Y1* State-Trait Anxiety Inventory—y form, *TO* time at the start of the session, *T1* time during the session, *T2* time at the end of the session. **(A)** Comparison between time T2 and time T0 of the STAI-Y score respectively within the music therapy group and in the control group. Music therapy group. *: T0 vs T2 = [42.00 (38.25–60.50) vs 34.50 (23.25–40.00); p = 0.000]. **(B)** Comparison between the time T2, T1 and T0 of the O2 Sat% respectively within the music therapy group and in the control group. Music therapy group. °: T0 vs T1 = [96.50 (95.00–97.75) vs 98.00 (97.00–99.00; p = 0.000]. °°: T0 vs T2 = [96.50 (95.00–97.75) vs 97.50 (96.25–99.00); p = 0.000]. Significance was assumed for p values < 0.050.

**Figure 2:**
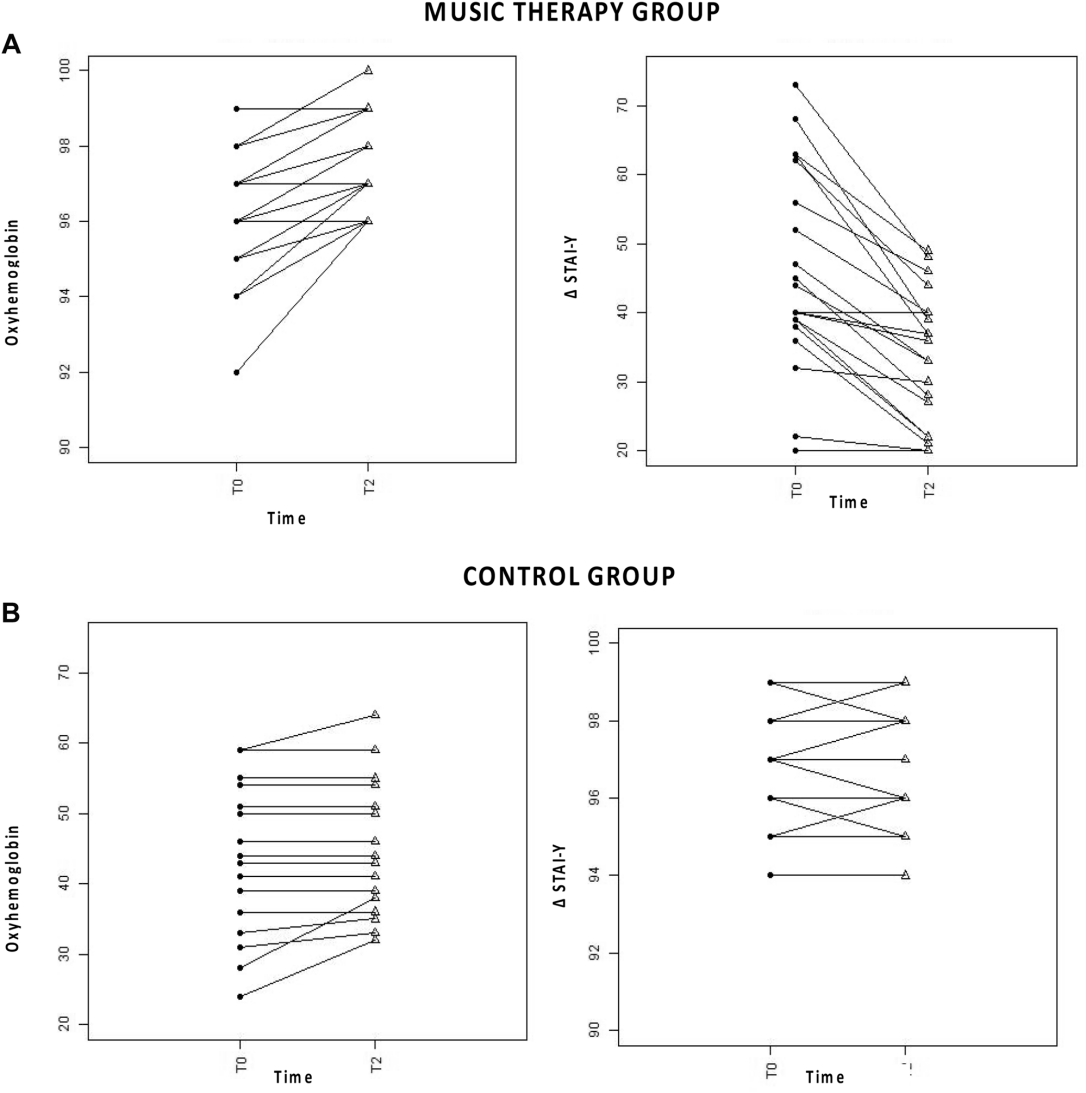
Intra-group comparison between the times T0 and T2. Trend of STAI-Y and oxyhemoglobinic saturation between the beginning and the end of the session in the 20 patients of the MG and in the CG. *STAI-Y1* State-Trait Anxiety Inventory—y form, *TO* time at the start of the session, *T1* time during the session, *T2* time at the end of the session, *STAI-y* State-Trait Anxiety Inventory—y form, *TO* time at the start of the session, *T1* time during the session, *T2* time at the end of the session. **(a)** MUSIC THERAPY GROUP. Intra-group comparison between T0 and T2 times regarding the variation of oxyhemoglobin saturation and STAY-Y. **(b)** CONTROL GROUP. Intra-group comparison between T0 and T2 times regarding the variation of oxyhemoglobin saturation and STAY-Y. *p < 0.050.

Within the group of patients undergoing receptive music therapy there was a significant reduction (p = 0.000) in the anxiety values in the period of time between the end of the music session at time T2 [34.50 (23.25–40.00)] compared to the beginning of the session at time T0 [42.00 (38.25—60.50)] (Figs. 1A, 2A). On the other hand, no difference emerged in CG.

As can be seen in Figs. 1B, 2B, in Group 1 the O2 Sat% after 10 min from the start of the music session (time T1) improved significantly statistically [T0 vs T1: 96.50) vs 98.00%; p = 0.000]. Similarly, at the end of the session (time T2) the significant increase in O2Sat was maintained compared to time T0 [T0 vs T2: 96.50% versus 97.50%; p = 0.000]. The same trend could be seen in HR.

### Discriminant analysis

**Figure 3:**
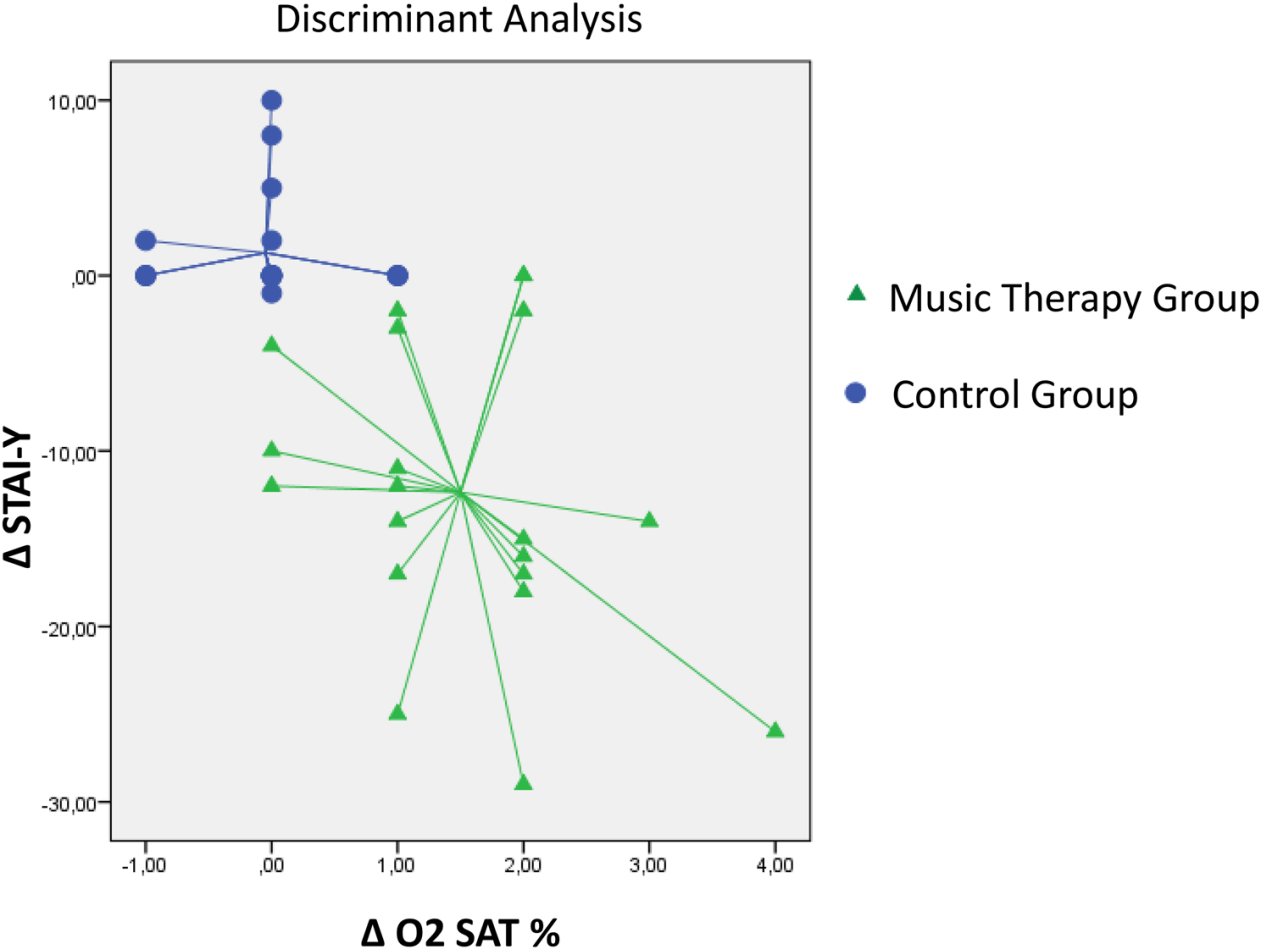
Discriminating analysis between the music therapy group and the control group. *STAI-y* State-Trait Anxiety Inventory—y form, *TO* time at the start of the session, *T1* time during the session, *T2* time at the end of the session, **Δ**STAI-Y: difference between the value of STAI-Y calculated at time T2 minus the value of **Δ**STAI-Y calculated at time T0; **Δ**O2 Sat%: difference between the value of O2 Sat calculated at time T2 minus the value of O2 Sat at time T0. The cross validate value able to discriminate the music therapy group compared to the control group starting from parameters **Δ**STAI-Y and **Δ** O2 Sat% was 90%.

The derived parameters ΔSTAI-Y and ΔO2 Sat were used to verify how much the improvement in anxiety and oxyhemoglobin saturation were able to discriminate the group undergoing music therapy compared to the control group. As can be seen from Fig. 2, 90% of the subjects were correctly classified within the two groups starting from the parameters ΔSTAI-Y and ΔO2Sat. There is no overlap between the two figures as can be seen graphically (Fig. 3).

## Discussion

This study supports the feasibility of introducing music therapy on site in Covid-19 patients as a supporting complementary/non-pharmacological intervention. Results show that a single session of receptive music therapy improves O2Sat and can significantly reduce anxiety.

All patients in MG completed the session of receptive music therapy and engaged openly with the music therapist, with interest and receptivity. No drop-out were registered. Despite the difficulties in speaking and interacting with patients due to personal protective equipment, music therapist was able to complete each session in safely and with acceptable discomfort. The receptive music therapy intervention did not prevent standard health procedures and did not hinder the work of health personnel, who proved to be available and collaborative.

Results show that MG patients, compared to CG, not only had significantly lower anxiety values in T2 than T0, but 70% had no more anxiety, and 30% had low anxiety values. Considering that anxiety can impose harmful effects on the course of recovery and overall well-being of Covid-19 patients, the significant reduction we found in anxiety suggests that music therapy was particularly helpful and highlights how the receptive music therapy intervention can support and contain the numerous stressful factors to which these patients are subjected.

Anxiety and stress affect and arouse sympathetic nervous system with numerous adverse responses including arterial and venous constriction, myocardial stimulation, and bronchoconstriction^15^. Music could have a positive effect on the para-sympathetic system, resulting in a relaxation response characterized by alpha brain wave frequency on the electroencephalogram, and physiologically manifesting itself as a state of muscular relaxation with regular deep breathing and lowered heart rate^16^. These physiological manifestations could be the reason why in MG there was a greater increase in O2Sat in T2 and T1 than T0. This data is important if we consider that increased breathing difficulty and fatigue were two of the most common symptoms in Covid-19 patients.

Previous studies on music therapy found improvements in anxiety, pain, quality of life, small positive effects on heart rate, respiratory rate, blood pressure, fatigue^17^ and positive changes on emotional state, social interaction, and spiritual well-being^18,19^. Furthermore, when compared to pharmacological sedation, music therapy is cost-effective, has no apparent risks and can provide patients and families with physical, emotional, and cultural benefits^20^. However, no studies have yet been conducted with Covid-19 patients in such a complex and difficult setting, both for patients and for the therapists themselves.

The emotional perception of music and its associated physical effects seem to involve catecholamines, but to be engaged in an music therapy session is first and foremost an experience with music and music therapist, and cannot be reduced to physical mechanisms alone. Its therapeutic potential must principally implicate other aspects characteristic of this experience—attitudes, expectations, affects, the imagination, memory, bodily self-awareness-^21,22^. Receptive music therapy technique used in this study, together with direct interaction with music therapist, helped the patient, stimulated by the music, to find and connect to internal resources of confidence, to cope with the present stress^13^ and to facilitate psychodynamic responses.

Using a music medicine intervention, patients would have been subjected to passive listening to pre-recorded music provided to the patient by a nurse or other medical staff, without being guided and accompanied to listen to the music. They would not have been able to share their experience with anyone and they would be alone again. Furthermore, music therapy intervention allows to adapt and modify play lists in real time, based on the verbal and non-verbal responses that patients have while listening.

Due to this, our intervention was made up of 5 closely related steps.

The strength of this approach was in systematically and specifically combining imagery and selected music by music therapist and patients. The listening experience involved thoughts, feelings, emotions and all the senses—visual, auditory, kinesthetic, gustatory, olfactory-.

As the surrounding environment was filled with artificial light and was distracting, patients were invited to close their eyes and to keep in touch with the images of their focus. All the patients who were involved found a focus with images from nature (sea, meadow, forest). The music prepared and selected by the music therapist then began and continued for about 15 to 20 min. While listening to the music, a dialogue between the patient and the music therapist was possible, encouraging patient’s self-exploration. The patient’s images that emerged while listening were guided, rather than controlled, by the music therapist. In the 5 step, the patient was able to validate his/her feelings, reinforcing the positive experience.

While listening to the first music track, many patients began to cry, then became reflective, sharing feelings, thoughts, and memories^9^. Patients reported how crying helped them release stress, anxiety and how they felt safe and secure crying along with the music. None of them stopped listening before the session ended. Within this specific approach to music, the presence of the music therapist could provide support, validating the patients’ own emotional states through active listening, attunement, and verbal acknowledgment of feelings expressed. Music and images allowed patients to live an experience beyond that of the ward, as if they were in another place, away from the worries and tiredness of the present situation. The music played the role of the co-therapist. In this complex period of emergency, care and human needs, when Healthcare workers are called to respond to the pandemic both clinically and humanely, complementary and interdisciplinary therapies, such as music therapy can offer a valid support, facilitating the expression of emotions and memories and strengthening patients’ self-awareness, social connection, and sense of personal support^22^. Thanks to this individual and customized intervention, patients in MG were able to express and communicate their feelings, shifting from an anxious and worried state, to feeling calm and content after the session. By the end of the session, many patients appeared soothed, displayed positive affect such as smiling and laughing, and shared appreciative comments about the session (Table 2).

**Table 2:** Comments provided from patients by the end of session.

1	It was a pleasant experience listening to the music. I felt different feelings and emotions depending on the rhythms, melodies and frequencies I listened to, I associated each track with a particular moment experienced here in the intensive care ward. Some music reminded me of the first phase, when all my thoughts were negative. Then, after going through the psychological and physical trauma that covid induces, the music moved towards recovery. The last track made me cry because I lost my parents to covid. The overall feeling was not of pain but of pleasant memories and associations. I imagined many scenes from nature
2	I think this is a really useful experience to find peace again. It offered essential psychological support for me. Even though I had my own earphones and access to my music, I had never been able, to reach this level of peacefulness since being here. Thank you
3	The music upset me a bit at first, but then it helped me reconnect with a part of myself
4	An incredible experience. I took a trip away from this place
5	It’s a very worthwhile experience, almost like being tele-transported to feelings and happy times in our past lives. It’s a mental and almost physical escape from a painful place, that does not affect your ability to react to such critical moments. Actually, it helps you to escape and distracts your mind from all the problems of the moment
6	Music is a wonderful thing
7	I like this kind of mental escape that you have with music because it helps you forget the negative things here and to think of a better future. On all fronts, from health, to life in general. Thank you … what a wonderful experience
8	The first thing I felt was curiosity. Imagination. It was like diving. The second track was amazing, relaxing, free… It was like flying like a butterfly, emptied out inside. I felt reborn, clean, completely free
9	It was wonderful to listen to this music… I ‘m crying with joy
10	I imagined myself riding my motorbike with my wife. It’s a really positive experience. I recommend it if you’re hospitalized here. It helps to escape
11	Life, love and music is about sharing with the people who matter to you. Being able to share the images and feelings you have with those close to you is definitely a great approach to the world and our lives
12	A sublime experience. From the first note the music gave me a sense of well-being accompanied by flashes of light. Then it sort of brought me energy. During the third song I thought of death, of sadness, but then I felt serene. At the end I felt reborn. The notes gave me strength, awareness, inner energy. I felt a little stronger. Thank you for letting me be a part of this experience. It made me feel intensely alive and peaceful
13	On the streets of New York, I imagined being in the middle of the skyscrapers and walking along the boulevards, seeing the shop windows and Italian restaurants… And talking to other Italians like me…
14	After such a long time, from death to life
15	Music not just for patients... I recommend it for the practitioners too
16	You are giving me so much joy. Thank you for the time you’ve given me and for this surprise
17	It was a positive adventure. Music relaxes you and makes you feel good in these moments
18	How wonderful. Thank you
19	How wonderful. A beautiful moment. There was the sea. I’ll always carry this moment with me; every time I’ll see the sea I’ll remember this moment. It gave me a little extra strength. Thank you
20	Thank you so much

## Limitations

It was not possible to enroll a greater number of participants and no placebo was used in the control group. Blinding of participants and researcher is not present. Allocation concealment was not done. Any long-term effects of our single receptive music therapy intervention were not evaluated. The duration of the anxiety-reducing effects cannot be determined. Since all of the participants were enrolled from the same hospital, their demographic characteristics might be somewhat similar and might not reflect the characteristics of the broader population. It is also possible that patients’ responses could have been biased due to the attention they were receiving from the researcher. Reports of better performance in post intervention questionnaires could have been biased and the Hawthorne effect cannot be excluded.

## Conclusions

This study provides important preliminary data that support and encourage the integration of music therapy into clinical practice. Despite the difficulties of working conditions (PPE) and despite the absence in literature of experiences of music therapy on site with covid patients, this study demonstrates the feasibility of introducing music therapy as an important form of support provided in unexpected and unpredictable extreme situations, when applied appropriately and judiciously.

The positive feedback of patients and the indication of a positive clinical impact would suggest the importance of further investigation into how music therapy can promote comfort and relaxation, lower anxiety, affect physiological outcomes and enhance psychosocial support (Suppl Information).

## Supplementary Information

Supplementary Information.
