# Supplementary figures and images for: Effect of single session receptive music therapy on anxiety and vital parameters in hospitalized Covid-19 patients: a randomized controlled trial
Source: Sci Rep. 2022 Feb 24;12:3154. doi: 10.1038/s41598-022-07085-8 (PMC8873232; doi:10.1038/s41598-022-07085-8)

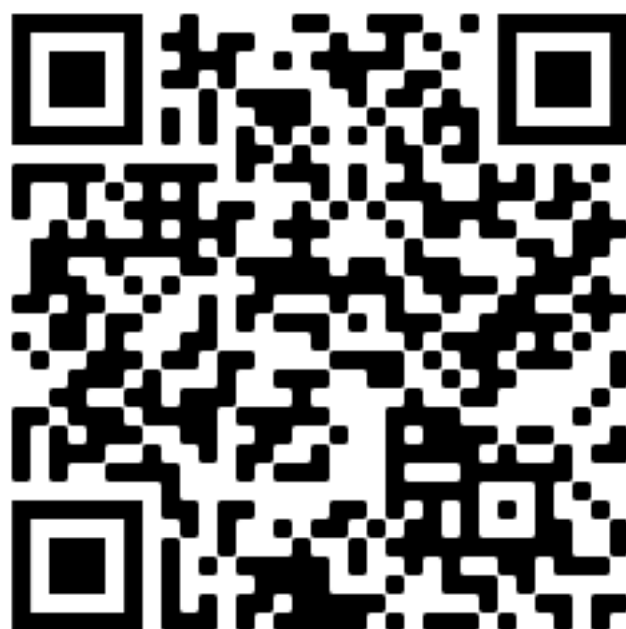

Supplement: Supplementary file 1 — Supplementary Information. [file 41598_2022_7085_MOESM1_ESM.pdf]
